# Cardiac Rehabilitation in Real Life

**DOI:** 10.1097/MD.0000000000001257

**Published:** 2015-08-14

**Authors:** Piotr Jankowski, Andrzej Pajak, Radoslaw Lysek, Anna Lukaszewska, Renata Wolfshaut-Wolak, Piotr Bogacki, Janusz Grodecki, Ewa Mirek-Bryniarska, Jadwiga Nessler, Piotr Podolec, Kalina Kawecka-Jaszcz, Danuta Czarnecka

**Affiliations:** From the First Department of Cardiology and Hypertension, Institute of Cardiology, Jagiellonian University Medical College (PJ, AL, KKJ, DC), Department of Clinical Epidemiology and Population Studies, Institute of Public Health, Jagiellonian University Medical College (AP, RWW, RL), Department of Cardiology, Ludwik Rydygier District Hospital (PB), Department of Cardiology, Gabriel Narutowicz Memorial General Hospital (JG), Department of Cardiology, Józef Dietl Hospital (EMB), Department of Coronary Heart Disease, Institute of Cardiology, Jagiellonian University Medical College (JN), Department of Cardiac and Vascular Diseases, Institute of Cardiology, Jagiellonian University Medical College (PP), Krakow, Poland.

## Abstract

Participation in cardiac rehabilitation programs (CRPs) improves prognosis in patients with coronary artery disease (CAD). However, not much is known about the effectiveness of CRP in real life. The aim of this analysis was to identify factors related to the referral to CRP following hospitalization for CAD and estimate the effectiveness of the programs in real life.

Medical records of 1061 consecutive patients aged ≤80 years, hospitalized due to an acute coronary syndrome or for a myocardial revascularization procedure in 5 hospitals serving the city and surrounding counties, were reviewed and 611 patients were interviewed 6–18 months posthospitalization.

Of 611 patients participating in the interview, 212 (34.7%) were referred following the hospitalization to a center providing CRP. Age, hospitalization in a teaching hospital, and index diagnosis were independently related to being granted a referral. Among the referred patients, 86.3% participated in the CRP. Participation in CRP was related to the lower probability of having high total cholesterol (23% vs 32%, *P* < 0.05), fasting glucose (11% vs 18%, *P* = 0.05), Hb_A1c_ (8% vs 16%, *P* = 0.05), and body mass index (27% vs 37%, *P* < 0.05). Generally, the effect of the CRP was significant in participants with a higher education, but not in those with a low education level. Other factors were not significantly related to the effectiveness of CRP.

This study shows that CRPs are effective, but underused in Poland. The participant's education level may influence the effectiveness of CRP. Therefore, in order to increase the impact of CRP, the content of such programs should vary depending on the education level of the participants.

## INTRODUCTION

Cardiovascular disease is the first cause of death worldwide.^1^ Patients with established coronary artery disease (CAD) are at high risk of recurrent cardiovascular events. In spite of the development in pharmacological and invasive treatment methods, risk factors remain independent predictors of cardiovascular mortality in CAD patients.^2^ The conclusion from the 5-year follow-up survey was that smoking cessation, providing dietary advice, and ensuring optimal pharmacological treatment are crucial in reducing mortality in patients who have suffered from a myocardial infarction.^3^ Thus, the highest priority for preventive cardiology was given to patients with established CAD.^4^

Participation in the cardiac rehabilitation and education program was found to be related to an improved lifestyle and better prognosis following an acute coronary event.^4–9^ However, less is known about the contemporary effectiveness of rehabilitation programs in real life. Moreover, not all patients participate in such programs.^8,10–12^ It was shown that the participation rate in rehabilitation programs following acute coronary syndromes in Poland is about 20%.^10^ It was also suggested that the participation rate may have even been decreasing in the recent years.^13^ Therefore, the aim of the present analysis was to identify factors related to the referral to cardiac education and rehabilitation programs following hospitalization due to CAD, as well as to estimate the effectiveness of the programs in real life.

## METHODS

Five hospitals with cardiology departments, serving the city and surrounding districts in the southern part of Poland, participated in the study. The total population of this area was about 1,200,000 inhabitants. In each department, the medical records of consecutive patients hospitalized from January 2010 to April 2012 due to acute myocardial infarction (first or recurrent, no prior percutaneous coronary intervention [PCI] or coronary artery bypass grafting [CABG]), unstable angina (first or recurrent, no prior PCI, CABG, or myocardial infarction), PCI (first, no prior CABG), or scheduled for CABG (first) were reviewed, and patients aged ≤80 years were identified retrospectively excluding those who had died during their in-hospital stays and those who were scheduled for CABG combined with valve surgery. If a patient was hospitalized more than once within the study period, only the first hospitalization was accepted as an index event. The medical records of patients fulfilling the inclusion criteria were analyzed using the standardized data collection forms.

Participants were invited to take part in the follow-up examination 6 to 18 months after discharge. Data on demographic characteristics, personal history of CAD, smoking status, blood pressure, fasting glucose, plasma lipids, and prescribed medications were obtained using a standardized data collection form. Patients’ height and weight were measured in a standing position without shoes and heavy outer garments using standard scales with a vertical ruler. Body mass index (BMI) was calculated according to the following formula: BMI = weight (kg)/height (m).^2^ Blood pressure was measured twice, on the right arm in a sitting position after at least 5 minutes of rest. For plasma lipid and glucose measurements, a fasting venous blood sample was taken between 7.30 and 8.30 am. For the present report, results of the analyses carried out no later than 4 hours after blood collection were used. All analyses were performed at one central laboratory. Low-density lipoprotein (LDL) cholesterol levels were calculated according to Friedewald formula. Carbon monoxide in the breath was measured for biochemical validation of self-reported nonsmoking. The concentration of breath carbon monoxide was recorded in parts per million using the Smokerlyser (Bedfont Scientific, Kent, UK).

We defined the patient as a participant of the cardiac rehabilitation and education program if he or she participated in at least half of the planned rehabilitation sessions.

We also calculated the secondary prevention coefficient: one point was awarded for each risk factor controlled (not smoking, blood pressure <140/90 mm Hg, LDL cholesterol <1.8 mmol/L, fasting glucose <7.0 mmol/L, BMI <25 kg/m^2^) during the follow-up interview. Additionally, one point was awarded for taking an antiplatelet agent, one point for taking an angiotesin-converting enzyme (ACE) inhibitor or a sartan, and one point for taking a β-blocker in patients with a history of heart failure or myocardial infarction. Thus, the secondary prevention coefficient could vary from 1 to 8. The survey protocol was approved by the Bioethics Committee of the Jagiellonian University.

### Statistical Analysis

Categorical variables were reported as percentages and continuous variables as means ± standard deviation. The Pearson χ^2^ test was applied to all categorical variables. Normally distributed continuous variables were compared by using the Student *t* test or analysis of variance. Variables without normal distributions were evaluated using the Mann–Whitney *U* test or the Kruskal–Wallis analysis of variance as appropriate. In order to find factors independently related to the probability of being granted a referral to the rehabilitation center, stepwise multivariate logistic analysis was performed. Assessing the relation between participation in cardiac rehabilitation and risk factor control, we constructed 2 multivariable models. Model 1 contained all variables independently related to being granted a referral to the rehabilitation center, whereas Model 2 (full model) contained also sex, education, employment, and practice setting. Finally, we performed subgroup analysis of the relation between participation in cardiac rehabilitation and the secondary prevention coefficient. A 2-tailed *P* value of <0.05 was considered as indicating statistical significance. In order to assess prevalence of risk factors, it was calculated that a sample of 500 patients, who attended for interview, was sufficient to estimate prevalences with precision of at least 5%, and with a confidence interval of 95%. We used the STATISTICA 8.0 software (StatSoft Inc., Tulsa, OK).

## RESULTS

The medical records of 1061 patients were reviewed and included in the analyses. Of 1061 hospitalized patients, 616 (58.1%) took part in the follow-up interview 6 to 18 months after discharge. Additionally, 5 patients could not decide (did not remember) whether they had or had not participated in a rehabilitation or education program following the index hospitalization. In consequence, we finally included the data of 611 patients in the present analysis. A possible selection bias in the formation of this study population was examined by comparing it with respect to age, sex, risk factors, and the prescription rate of drugs upon discharge with 450 patients on whom we had no data concerning participation in a rehabilitation program. These comparisons did not reveal any statistically significant differences with respect to all the above factors except for age at the time of hospitalization (63.6 ± 8.8 years in patients participating in the interview vs 64.9 ± 10.3 years in nonparticipants; *P* < 0.05) and the prescription rate of ACE inhibitors/sartans upon discharge (88.0% in participants vs 82.9% in nonparticipants; *P* < 0.05). We also compared the attendance rates between the index event groups showing a slight but statistically significant bias (*P* < 0.05), characterized by a somewhat higher attendance rate in the PCI group (53.8%, 61.0%, 65.3%, and 50.9% for myocardial infarction, unstable angina, PCI, and CABG group, respectively).

The mean period of time from the discharge to the follow-up interview was 1.1 ± 0.2 years. Of 611 patients participating in the follow-up interview, 212 (34.7%) were referred to a center providing a cardiac rehabilitation program (CRP). The characteristics of patients referred and not referred are presented in Table 1. Age, hospitalization at a teaching hospital, and index diagnosis were independently related to being granted a referral (Table 2). Of 611 patients, 184 (30.1%) participated in at least half of the planned rehabilitation sessions. Among the referred patients, 86.3% participated in the rehabilitation program. When we analyzed the whole study, population age, hospitalization in a teaching hospital, and index diagnosis were independently related to participation in the rehabilitation program (Table 2). However, when the referral was included in the statistical model, we found that only 2 factors were independently related to participation in the cardiac rehabilitation, that is, the referral (odds ratio [OR] 2514, confidence intervals [CI]: 330–19,169), and CABG (OR 6.6, CI; 1.12–37.1). In patients referred to rehabilitation, only 1 factor—CABG as an index event—was significantly related to participation in a rehabilitation program (OR 8.3, CI: 1.1–64.9).

**TABLE 1:**
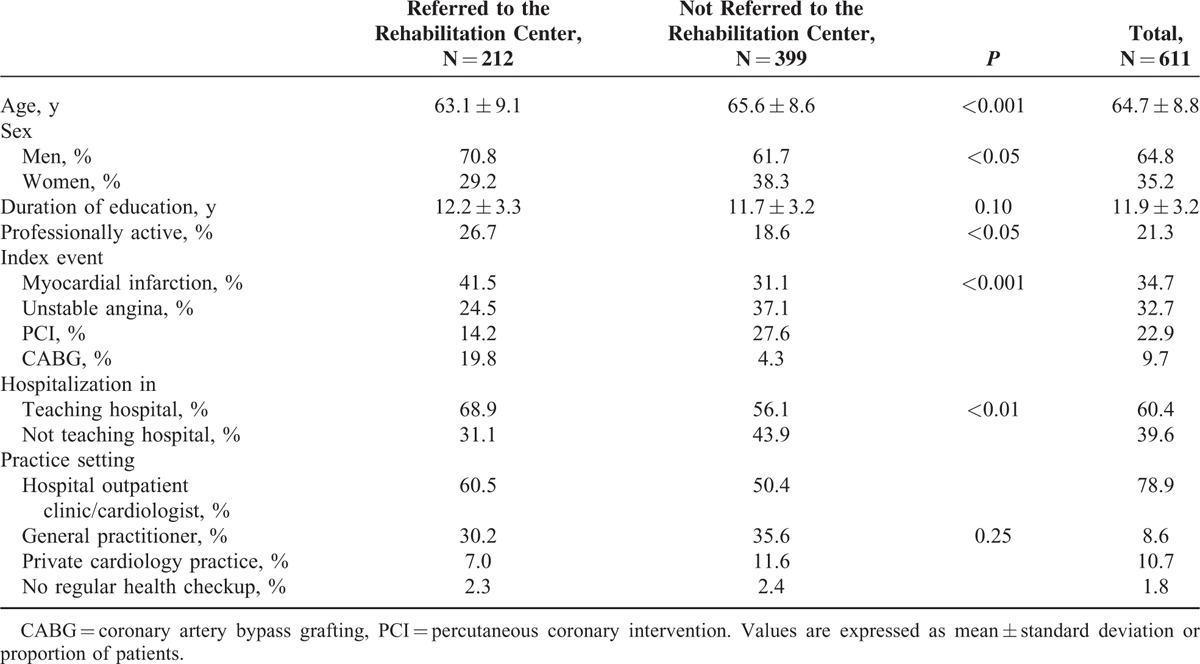
Characteristics of the Study Group

**TABLE 2:**
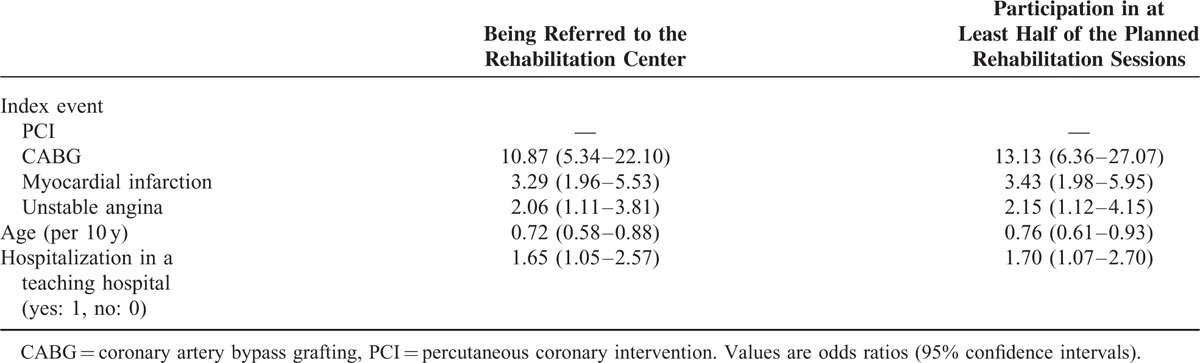
Variables Independently Related to the Probability of Being Granted a Referral to the Rehabilitation Center Following Hospitalization Due to Coronary Artery Disease and to the Probability of Participation in at Least Half of the Planned Rehabilitation Sessions (N = 611)

Participation in the rehabilitation program was related to a lower mean BMI and fasting glucose, Hb_A1c_, and total cholesterol (Table 3). A significantly lower proportion of patients who had participated in a rehabilitation program had high BMI, total cholesterol, fasting glucose, and Hb_A1c_ (Table 4). Participants of the rehabilitation program were less frequently prescribed diuretics and calcium antagonists (Table 5); however, the latter association was not significant in multivariate models (Table 6).

**TABLE 3:**
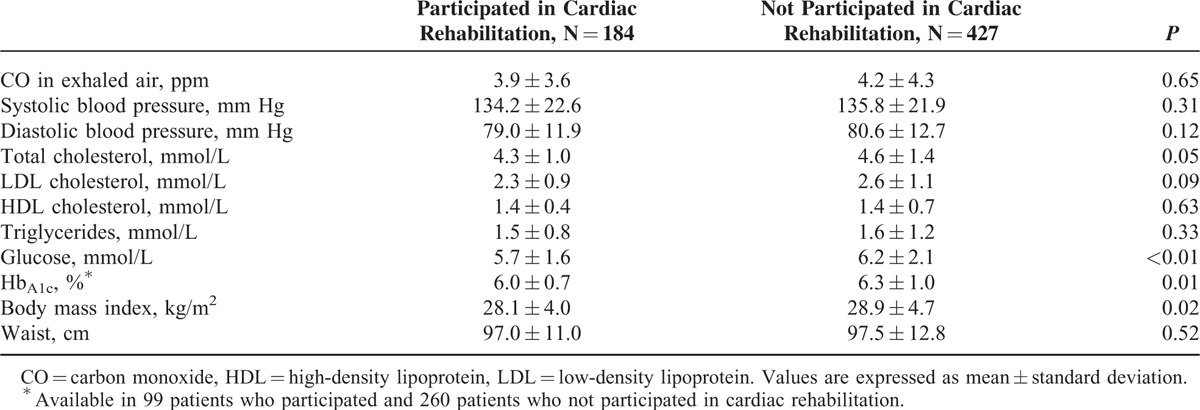
Relation Between Participation in Cardiac Rehabilitation and the Level of Risk Factors 6–18 mo After Discharge

**TABLE 4:**
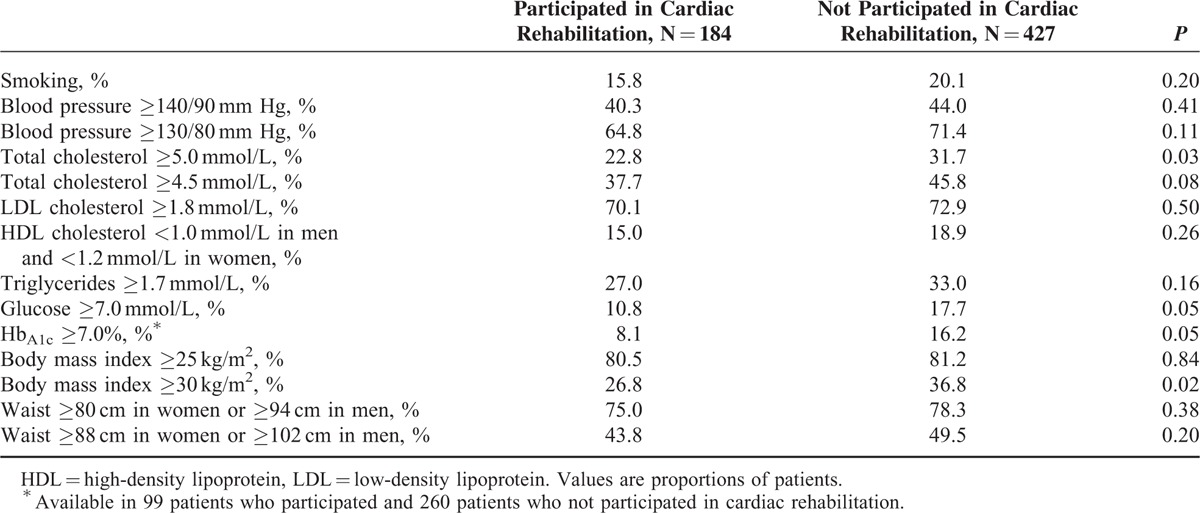
Proportions of Patients Participating and Not Participating in Cardiac Rehabilitation With Uncontrolled Risk Factors 6–18 mo After Discharge

**TABLE 5:**
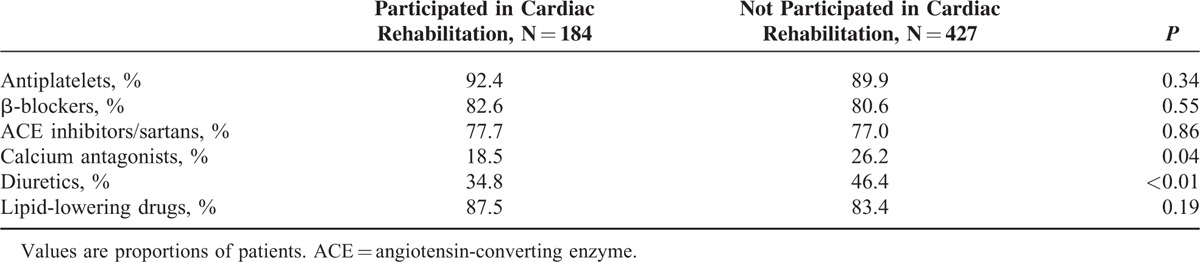
Proportions of Patients Participating and Not Participating in Cardiac Rehabilitation Taking Cardiovascular Drugs 6–18 mo After Discharge

**TABLE 6:**
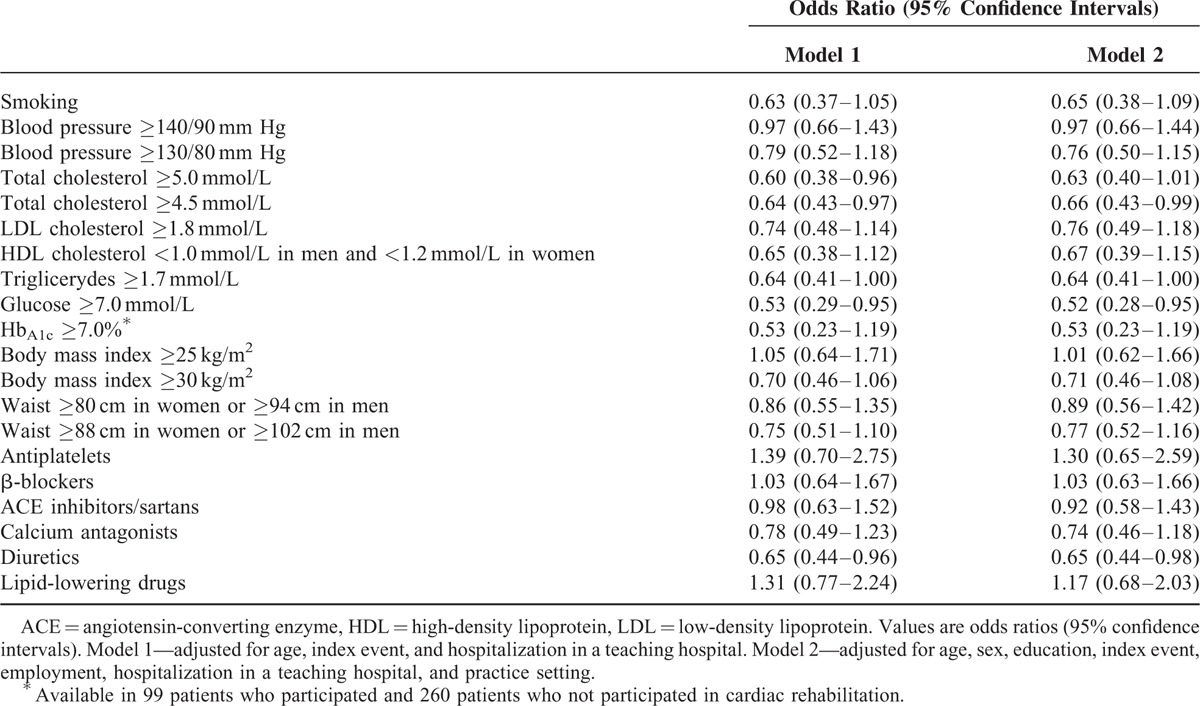
Odds of Noncontrolled Risk Factors and Drugs Use 6–18 mo After Discharge Related to Participation in Cardiac Rehabilitation

The mean of the secondary prevention coefficient was 5.26 ± 1.32. The coefficient was significantly higher in patients who had participated in a rehabilitation program (Table 7). There was significant interaction between participation in the program and education, that is, the difference was significant in participants with a higher education, but not in those with a low education level (Table 7).

**TABLE 7:**
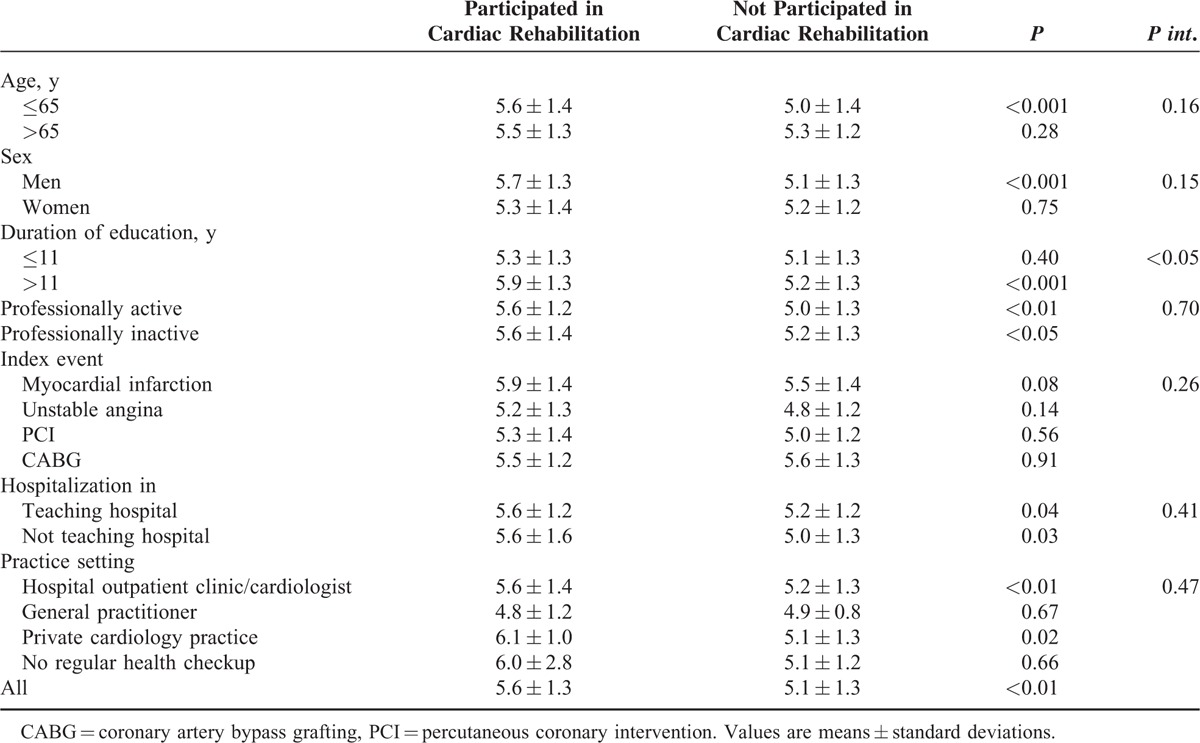
Mean Values of the Secondary Prevention Coefficient in Subgroups of Patients Participating and Not Participating in Cardiac Rehabilitation

## DISCUSSION

In general, our results showed a considerable potential for the further reduction in cardiovascular risk following hospitalization for CAD. Indeed, cardiac rehabilitation and education programs improve prognosis following an acute coronary event.^3–5,14^ Importantly, only 35% of our study participants were referred for cardiac rehabilitation and only 30% participated in at least half of the planned rehabilitation sessions. These numbers are similar to cardiac rehabilitation utilization in other countries.^15–16^ Low participation rate could suggest that the easiest way to improve prognosis after hospitalization for CAD is to increase access to cardiac rehabilitation and education programs. The lower referral rate among older patients could be partly explained by relatively lower evidence of benefit in this age group compared to younger patients.^17^ However, it should be underlined that physical activity ameliorates cardiovascular health also in elderly patients.^18^

Usually, the decision about the referral to a center providing CRPs is made by the physician whereas the decision about participation in the program is made by the patient (with or without his/her family). We noted that 86.3% of referred patients participated in the cardiac rehabilitation. Nevertheless, we looked separately for factors related to referral and to participation in the rehabilitation and we found similar factors related to being referred and to participation in the CRP. Indeed, our results suggest that the decision of the physician is crucial. Our study showed a considerable selection bias in the profile of patients who were advised to attend a CRP. Interestingly, among all index events, the PCI was significantly related to the lower probability of being referred to a rehabilitation center (Table 2). Importantly, the potential gain from cardiac rehabilitation is considerable irrespective of the diagnostic group.^14^ In contrast to the present evidence, results from the Euroaspire III survey suggested that patients hospitalized for acute myocardial ischemia (troponine negative) have the lowest probability of being referred to a rehabilitation center.^15^ Another important difference is the relation between the patient's education level and the probability of being referred to a rehabilitation center.^15^ It should, however, be stressed that participants of the Euroaspire III survey were hospitalized about 6 to 7 years earlier as compared to the participants of the present study. Indeed, the physicians’ approach to cardiac rehabilitation could change favorably with time. Another explanation could be the different approach in Poland compared to most other European countries. In contrast to the Euroaspire III survey, we were able to analyze the relation between teaching versus regular hospitals and we could show that patients hospitalized in teaching hospitals were more frequently referred to rehabilitation centers.

The term cardiac rehabilitation refers to coordinated, multifaceted interventions designed to optimize a cardiac patient's physical, psychological, and social functioning, in addition to stabilizing, slowing, or even reversing the progression of the underlying atherosclerotic processes, thereby reducing morbidity and mortality.^19^ Core components of modern cardiac rehabilitation and education programs include comprehensive lifestyle intervention in relation to stopping smoking, making healthy food choices, and becoming physically active, as well as weight, blood pressure, lipids and glucose management, and psychosocial support.^4,5^ Indeed, it was suggested that CRPs should evolve into cardiovascular risk reduction programs instead of being concentrated on exercises.^20^

Although we found a significant difference in the secondary prevention coefficient between those who had and those who had not participated in cardiac rehabilitation, a greater difference could be expected. However, about 20% of all Polish participants of the Euroaspire III survey reported that the rehabilitation program contained a dietary modification aspect and only 18% of smoking Polish participants reported that the rehabilitation program aimed at the cessation of smoking.^15^ One could expect a positive correlation between the risk factor control and the quality of the CRP. Indeed, it seems that there is a room for the further reduction of cardiovascular risk following hospitalization for CAD through improvement in the quality of CRPs in Poland.

The significant relationship between the secondary prevention coefficient value and the participation in cardiac rehabilitation was shown previously.^13^ The results of the present analysis suggest that the effectiveness of cardiac rehabilitation is significantly greater in higher educated patients. We were not able to find any other significant interactions. Our results suggest that the content of the education and rehabilitation program should depend on the participants’ education and that more focus on patients with a low education is needed.

It is possible that patients after a severe cardiac event may be more susceptible to education. Indeed, this could influence the effects of the CRP. Unfortunately this is not supported by our results. Indeed, the mean value of secondary prevention coefficient between CABG and PCI group was not higher among those who had participated compared to patients who had not participated in the cardiac rehabilitation. Moreover, no such phenomenon can be found when patients from myocardial infarction and unstable angina groups are compared suggesting that other factors could influence the lack of the rehabilitation effect in the CABG group.

### Limitations of the Study

Interpretations of the results of the present study have some limitations. First, it is possible that some unrecognized differences exist between patients who had and those who had not participated in the cardiac rehabilitation. These differences could influence the approach to secondary prevention in the study participants. Second, we were unable to assess the impact of the differences in the implementation of secondary prevention on the risk of cardiovascular events. Third, our study participants were not representative of all CAD patients. Participants were limited to those who had undergone an acute CAD event or revascularization procedure. Therefore, our results should not be directly applied to the other patients. Fourth, information on the referral and participation were obtained from self-reports. However, an important strength of our analysis is that it is not just based on abstracted medical record data but face-to-face interviews and examinations using the same protocol and standardized methods and instruments, including central laboratory analyses of lipids and glucose. Therefore, this analysis provides contemporary information on lifestyle, risk factor, and therapeutic management for secondary prevention. Fifth, secondary prevention coefficient was calculated as the number of recommended by the guidelines goals achieved.^4^ However, the severity of risk factors at baseline beside the quality of the medical service could have influenced the risk factors control.^21^ It should be also underlined that genetic background could influenced the risk factors control.^22–24^ Finally, it should be stressed that the differences in risk factor management at the interview should be interpreted in the context of selection bias with regard to advice and participation in cardiac rehabilitation, as it is unclear how far they are related to the effectiveness of the rehabilitation program or to the selection of patients in the respective groups.

## CONCLUSION

Using the data of consecutive patients hospitalized for CAD, we were able to show that CRPs seems to be effective and underused in real life. Our results suggest that the level of education of participants may influence the effectiveness of CRPs. Therefore, in order to increase the impact of cardiac rehabilitation and education programs, it should be considered to vary the content of such programs depending on the education level of the participants. Our results suggest that such programs should be more focused on patients with the low education level.
